# Pharmacogenetic testing affects choice of therapy among women considering tamoxifen treatment

**DOI:** 10.1186/gm280

**Published:** 2011-10-04

**Authors:** Wendy Lorizio, Hope Rugo, Mary S Beattie, Simone Tchu, Teri Melese, Michelle Melisko, Alan HB Wu, H Jeffrey Lawrence, Michele Nikoloff, Elad Ziv

**Affiliations:** 1 Division of General Internal Medicine, Department of Medicine, University of California San Francisco, 1545 Divisadero Street, Suite 322, San Francisco, CA 94143-0320, USA; 2 Division of Clinical Pharmacology and Experimental Therapeutics, Department of Medicine, University of California San Francisco, San Francisco General Hospital Medical Center, 1001 Potrero Avenue, Building 30, 2nd Floor, Room 3216, San Francisco, CA 94143-1220, USA; 3 Helen Diller Family Comprehensive Cancer Center, University of California San Francisco, 1600 Divisadero Street, San Francisco, CA 94143, USA; 4 Division of Hematology and Oncology, Department of Medicine, University of California San Francisco, 1600 Divisadero Street, Room B-608, San Francisco, CA 94143-1710, USA; 5 Department of Epidemiology and Biostatistics, University of California San Francisco, 185 Berry Street, Lobby 5, Suite 5700, San Francisco, CA 94107, USA; 6 Institute for Human Genetics, University of California San Francisco, 513 Parnassus Avenue, Suite S965, San Francisco, CA 94143-0794, USA; 7 Department of Biopharmaceutical Sciences, University of California San Francisco, 1700 Fourth Street, Byers Hall, Suite BH-216, San Francisco, CA 94143-0775, USA; 8 Department of Medicine, School of Medicine, Dean's Office, University of California San Francisco, 513 Parnassus Avenue, Medical Science Building 224, San Francisco, CA 94143-0410, USA; 9 Department of Laboratory Medicine, University of California San Francisco, 1001 Potrero Avenue, SFGH 5 2M27, San Francisco, CA 94143, USA; 10 Roche Molecular Systems, Inc., 4300 Hacienda Drive, Pleasanton, CA 94588, USA

## Abstract

**Background:**

Pharmacogenetic testing holds major promise in allowing physicians to tailor therapy to patients based on genotype. However, there is little data on the impact of pharmacogenetic test results on patient and clinician choice of therapy. *CYP2D6 *testing among tamoxifen users offers a potential test case of the use of pharmacogenetic testing in the clinic. We evaluated the effect of *CYP2D6 *testing in clinical practice to determine whether genotype results affected choice of hormone therapy in a prospective cohort study.

**Methods:**

Women planning to take or currently taking tamoxifen were considered eligible. Participants were enrolled in an informational session that reviewed the results of studies of *CYP2D6 *genotype on breast cancer recurrence. *CYP2D6 *genotyping was offered to participants using the AmpliChip CYP450 Test. Women were classified as either poor, intermediate, extensive or ultra-rapid metabolizers. Results were provided to clinicians without specific treatment recommendations. Follow-up was performed with a structured phone interview 3 to 6 months after testing to evaluate changes in medication.

**Results:**

A total of 245 women were tested and 235 completed the follow-up survey. Six of 13 (46%) women classified as poor metabolizers reported changing treatment compared with 11 of 218 (5%) classified as intermediate, extensive or ultra-rapid metabolizers (*P *< 0.001). There was no difference in treatment choices between women classified as intermediate and extensive metabolizers. In multi-variate models that adjusted for age, race/ethnicity, educational status, method of referral into the study, prior knowledge of *CYP2D6 *testing, the patients' *CYP2D6 *genotype was the only significant factor that predicted a change in therapy (odds ratio 22.8; 95% confidence interval 5.2 to 98.8). Genetic testing did not affect use of co-medications that interact with *CYP2D6*.

**Conclusions:**

*CYP2D6 *genotype testing led to changes in therapy among poor metabolizers, even in the absence of definitive data that an alternative medicine improved outcomes. Pharmacogenetic testing can affect choice of therapy, even in the absence of definitive data on clinical impact.

## Background

Pharmacogenetics may improve health outcomes by allowing clinicians to tailor medications to patients' individual genetic profiles. Once the genetic determinants of drug response are identified, additional work will be required to translate these findings into practice [1-3]. One major question regarding the implementation of pharmacogenetic testing is how clinicians will incorporate the results into practice and whether the genotypic results will lead to a change in therapy.

Tamoxifen, a selective estrogen receptor modulator, acts as an estrogen receptor antagonist in breast tissue. In the adjuvant setting, tamoxifen reduces breast cancer recurrence [4] and mortality [5,6] among women with hormone receptor-positive breast cancer. Tamoxifen also reduces the risk of breast cancer in high risk women [7]. It is metabolized to 4-hydroxy-N-desmethyl-tamoxifen, also known as endoxifen [8-10], which is considered the primary pharmacologically active metabolite of tamoxifen [9-11]. Cytochrome P450 2D6 enzyme (*CYP2D6*) is the rate-limiting enzyme that converts N-desmethyl-tamoxifen into endoxifen [10,12,13].

The *CYP2D6 *gene is highly polymorphic and has several alleles that decrease or completely abolish its enzymatic activity. Several studies suggest that breast cancer patients on tamoxifen with a 'poor metabolizer' phenotype (two inactive alleles) [11,14-18] or with two alleles with reduced enzymatic activity [16,19-24] have a higher rate of breast cancer recurrence compared to patients with other phenotypes. Recent retrospective analyses from two large randomized trials comparing tamoxifen with aromatase inhibition as treatment for early stage breast cancer in post-menopausal women demonstrated no impact of *CYP2D6 *genotype on outcome [25,26]. Nonetheless, the impact of genotype on the effectiveness of tamoxifen remains uncertain [11,13-23,27,28].

Although there is considerable controversy regarding the predictiveness of *CYP2D6 *genotypes on outcomes, there are alternatives to tamoxifen treatment. Aromatase inhibitors (AIs) are considered more effective at reducing breast cancer recurrence than tamoxifen alone in post-menopausal women with hormone receptor-positive breast cancer [29-33], although no impact has been demonstrated on mortality. In pre-menopausal women with early stage hormone receptor-positive breast cancer, tamoxifen with or without ovarian suppression (OS) remains the preferred treatment for standard adjuvant therapy since no current data demonstrate improved outcomes of pre-menopausal women on AIs plus OS [34,35]. However, OS alone or with AIs in pre-menopausal women may be considered an alternative in pre-menopausal women who do not tolerate tamoxifen [35-37]. Therefore, *CYP2D6 *testing may be considered a useful test case of the use of pharmacogenetic testing in the clinic since there are alternative treatments.

We prospectively evaluated the effect of *CYP2D6 *testing in clinical practice and the impact of providing genotype to practitioners and patients in a prospective cohort study. Specifically, we recruited women who had recently started or were considered candidates to start tamoxifen. They were offered *CYP2D6 *genotype testing and results were sent to the participant's clinician. We then followed women who underwent testing to determine whether the genotypes affected choice of therapy.

## Materials and methods

### Study population

Potential participants included women who were currently on tamoxifen or who were considered candidates for tamoxifen, either for treatment or prevention of breast cancer. Patients were recruited by physician referral or after receiving a contact letter sent to all patients from the University of California San Francisco (UCSF) Breast Oncology Clinic who met eligibility criteria. Participants were excluded if they could not give informed consent or could not participate in the educational session due to limited English proficiency. Recruitment took place between March 2008 and May 2010. Most of the women, 222, were referred to the study from physicians' offices. Of these, 15 (7%) did not agree to participate, leaving 207 (93%) referred women who consented to the study. Another 194 women were contacted by letter. Of those, 102 (52%) did not respond, 54 (28%) said they were not interested (*n *= 34) or not on tamoxifen (*n *= 20), leaving 38 (20%) women who were recruited by letter. Thus, a total of 245 women consented to participate in this study. The institutional review board at UCSF approved the study and all women provided written informed consent at study entry.

### Study protocol

Prior to attending the educational session, each participant was required to identify a referring physician. The referring physician received a short description of the study and agreed to receive the test result in order for the patient to be enrolled. After signing informed consent, the women participated in an educational session conducted by a study physician who used an oral and slideshow presentation to explain genetic testing in general. The study physician also showed slides that included both positive and negative studies regarding *CYP2D6 *genotype and breast cancer recurrence. The studies discussed included those published prior to March 2008 when recruitment began. The study physician explicitly told participants that genetic testing remains controversial in the medical literature and that additional studies of the utility of genetic testing on clinical outcome were underway. The presentation was approximately 30 to 45 minutes long, including 30 standardized slides and time for questions and discussion. Participants were asked to complete pre- and post-session questionnaires. *CYP2D6 *testing was offered to all participants at the end of the session (see laboratory protocols) and blood was obtained immediately after the educational component concluded. Results were released to the referring clinician 2 to 4 weeks after testing.

Follow-up was performed with a structured phone interview 3 to 6 months after test results were provided to physicians and patients to determine whether a change in medication occurred.

### Demographic, breast cancer risk factors and tamoxifen data collection

The pre- and post-educational session questionnaires collected the following information: demographics, past medical history, breast cancer history (including pathology and prior treatment), tamoxifen use, other co-medication use, knowledge of genetic testing, and attitudes towards uptaking new technology. Women were classified as pre-menopausal if they indicated having a menstrual period in the prior 3 months and no change in menstrual regularity in the prior year; they were considered post-menopausal if they had no vaginal bleeding (amenorrhea) for at least 6 months without other obvious pathological or physiological cause. Participants were asked if they were experiencing hot flashes, vaginal dryness, sleep problems and any other side effects from tamoxifen. The number, intensity, duration, and severity of hot flashes were reported in the questionnaire. Severity of each side effect was rated on a Likert scale with responses ranging from 1 (mild) to 5 (extremely severe).

### Laboratory procedures

If the participant agreed to testing, two 10 cc tubes of blood were drawn. One tube of blood was used for genomic DNA extraction that was performed at the UCSF Clinical Pharmacogenomics Laboratory. DNA was extracted from whole blood using the Qiagen QIAamp Blood DNA Kit (Frederick, MD, USA). After extraction, DNA was quantified and stored at -20°C. A second blood sample was collected in a serum separator tube and stored at -20°C to measure tamoxifen metabolites, especially endoxifen levels. Tamoxifen metabolite measurements were not reported to patients or clinicians since there were no clinical data on their use at the time the study was conceived and designed.

### *CYP2D6 *genotype

The analysis of *CYP2D6 *polymorphisms was performed at the UCSF Clinical Pharmacogenomics Laboratory, a Clinical Laboratory Improvement Amendments Act (CLIA)-certified laboratory, using the AmpliChip CYP450 Test (Roche Molecular Systems, Inc., Branchburg, NJ, USA). This test uses the Affymetrix microarray platform and screens for 27 different alleles of the *CYP2D6 *gene (including gene duplications and deletions) and 3 alleles of the *CYP2C19 *gene. The AmpliChip CYP450 Data Analysis Software was used to infer the genotype, and to predict the individual's *CYP2D6 *enzymatic activity. We classified subjects into four classes: ultra-rapid metabolizers (UMs), extensive metabolizers (EMs), intermediate metabolizers (IMs), and poor metabolizers (PMs). The test and assay conditions for this study followed the manufacturer's instructions [38]. In approximately 1 to 2% of samples, the test results in a 'no genotype' call, presumably because of a rare variant not detected by the chip that interferes with the usual hybridization patterns. In every case of a 'no genotype' result from the AmpliChip, we repeated the assay at least once to confirm that the result could not be obtained.

### Reporting of results

Clinicians were informed of test results, including the specific genotype and metabolizing status but no specific treatment recommendation was provided. Results were reported with the specific genotype (for example, *1/*4) and the interpretation of the enzymatic activity as classified by the AmpliChip CYP450 Test (for example, 'ultra-rapid metabolizer', 'extensive metabolizer', 'intermediate metabolizer', or 'poor metabolizer'). We used Table 2 from the AmpliChip package insert for the assignment of ultra-rapid, extensive, intermediate and poor *CYP2D6 *metabolizers. In addition, information about the effect of metabolizer status on endoxifen levels and the effect of co-medications was provided based on a commonly used reference [39]. Clinicians were not provided specific input about the relationship between genotype or metabolizer status and breast cancer recurrence because of the controversial nature of this association. Clinicians were provided with a form letter to help with informing patients that offered two possible recommendations: (a) to continue current therapy or (b) to call the clinician and schedule an appointment to discuss the results. The *CYP2C19 *genotypes from the AmpliChip test and endoxifen levels were not part of the main study and these results were not, therefore, reported to attending oncologists.

### Clinical follow-up

Three to six months after *CYP2D6 *testing, a follow-up questionnaire was administered by a trained research assistant during a structured telephone interview. This questionnaire ascertained whether the patients received the *CYP2D6 *test result letter and discussed *CYP2D6 *phenotype status (UM, EM, IM, PM) with their clinician, whether the clinician suggested any change in medication based on the test result (tamoxifen, AIs, or any other medication), and what change was suggested. We also determined whether the patients were still taking, started taking, or stopped taking tamoxifen since study participation and what the reason was for any change in hormone therapy.

### Statistical analysis

To evaluate the effect of *CYP2D6 *testing in clinical practice and to determine whether reported *CYP2D6 *phenotype affects change in therapy, we compared the rate of medication change among women identified as PM to women identified as UM, EM or IM using Fisher's exact test. In our analysis, data from women with UM and EM phenotype was combined into one category (UM/EM) since all reports suggest that they have the same clinical outcome. All analyses were conducted with the program STATA (version 10, StataCorp LP, College Station, TX, USA).

## Results

A total of 245 women were enrolled in the study, of whom 235 (96%) participated in the follow-up survey. Ten women (4%) did not return letters or telephone calls and were not included in the analysis of follow-up. The average age of women enrolled in the study was 47 years (range from 23 to 82; Table 1). Most of the participants were Caucasian (68%) and Asian (23%). Thirty-eight percent of women had other chronic health problems. Seventy-two percent of women were married. Educational attainment and income were high; 43% had completed post-graduate degrees and 44% lived in households with > $100, 000 income. At the time of breast cancer diagnosis, 78% (184) were pre-menopausal and 22% (51) were post-menopausal. Nearly all of the women enrolled in the study (97%) had either invasive breast cancer or ductal carcinoma *in situ *(DCIS) with the majority (70%) reporting invasive breast cancer.

**Table 1:** Demographics, breast cancer, tamoxifen use and co-medications use characteristics in the overall population in the study

Characteristics (*N *= 245)	N/mean	Percent/SD
**Mean age (years)**^**a**^	47.46	± 9.7
**Self-report ethnicity**		
Caucasian	166	67.76
Asian/East Asian	56	22.86
African American/Black	2	0.82
Latina/Hispanic	14	5.71
Pacific Islander	1	0.41
Other/mixed	3	1.22
Declined/refused/do not know	3	1.22
**Number married (yes)**	176	72
**Number full-time working**	98	40
**Education levels**		
High school graduated or less	6	2.45
Some college	36	14.69
College graduated	90	36.73
Completed post-graduate degree	105	42.86
Declined/refused	8	3.27
**Socio-economic status**		
Income < $50, 000	29	11.84
Income ≥$50, 000 to < $100, 000	56	22.86
Income ≥$100, 000	108	44.07
Declined/refused	52	21.23
**Reported other health problems**	91	38
**Breast cancer characteristics**		
Breast cancer (yes)	237	97
Had invasive breast cancer	165	70
Surgery (yes)	231	98
Had lumpectomy	119	52
**Menopausal status at diagnosis**		
Pre-menopausal	184	78
Post-menopausal	51	22
**Mean age at menopause (years)**^**a**^	45.61	± 6.79
**Had natural menopause**	35	22.73
**Menopause due to chemotherapy treatment**	74	48.05
**Previous used of hormone therapy**	37	15
**Tamoxifen use**		
Ever prescribed	191	78
Ever taken	171	70
Currently taking	166	68
**Common side effects attributed to tamoxifen**		
Hot flashes	154	63
Sleep problems	113	46
Vaginal dryness	90	37
**Co-medications/*CYP2D6 *inhibitors**		
**Strong inhibitors**		
Paroxetine	1	0.41
Bupropion	8	3.26
**Moderate inhibitors**		
Sertraline	8	3.26
Duloxetine	3	1.22
**All other inhibitors**		
Amitriptyline	2	0.82
Amlodipine	2	0.82
Celecoxib	2	0.82
Ceterizine	2	0.82
Citalopram	6	2.45
Diphenhydramine	3	1.22
Escitalopram	6	2.45
Imipramine	1	0.41
Loratadine	3	1.22
Nortriptyline	1	0.41
Ranitidine	1	0.41
**Other co-medications**		
Gabapentin	10	4.00
Trazodone	2	0.82
Venlafaxine	15	6.12
**Referral method**		
Physician/nurse referral	196	80
Self-referred or breast cancer support group referral	11	4
Study contact letter	38	16
**Previous knowledge of *CYP2D6 *testing (yes)**	122	50
**Source of *CYP2D6 *testing knowledge**		
Physician or nurse	46	38
Newspaper	5	4
Television	1	1
Internet	17	14
Medical literature	24	20
Other	27	22
Unknown/missed	2	1

^a^Data presented as mean ± SD. N, number of participants in the study; SD, standard deviation.

Sixty-eight percent (166) of women in the study were taking tamoxifen at the time of enrollment for a median duration of 5 months (range from 1 to 60). The most common side effects attributed to tamoxifen were hot flashes (63%), sleep problems (46%) and vaginal dryness (37%). Approximately 10% of women (24) in the study reported taking selective serotonin reuptake inhibitors (SSRIs), but only one was taking an SSRI considered to be a strong inhibitor of *CYP2D6 *(paroxetine). In addition, eight participants (3%) were taking a moderate to potent inhibitor, the norepinephrine-dopamine inhibitor buproprion.

The primary referral method in the study was by a physician or nurse (80%). The rest of the participants were either self-referred or referred by a breast cancer support group (4%) or recruited by the study contact letter (16%). Approximately 50% (122) of the women in the study had previous knowledge of *CYP2D6 *testing and the main source of this knowledge was a physician or nurse (38%). Other sources of prior knowledge regarding testing included women who reported reading about *CYP2D6 *in the medical literature (20%), the internet (14%), and television or newspapers (5%).

Table 2 shows the detailed *CYP2D6 *genotypes and predicted phenotype frequency distribution of participants in the study by ethnicity. Of the 245 participants, 4% (10) were UMs, 76% (185) were EMs, 13% (32) IMs and 5% (13) were PMs. In addition, in four of the women (2%), we could not ascertain the genotype based on the AmpliChip result (Table 2). Of the 13 PMs, 10 (77%) were Caucasian, 2 (15%) were Latina and 1 (8%) was Asian. There was no significant difference in the rate of PMs across these racial/ethnic categories. Of the 32 IMs, 15 (47%) were Asian, 15 were Caucasian and 2 (6%) were Latina. Asians were more likely to be classified as IMs compared to Caucasians (*P *= 0.002). Out of 166 women taking tamoxifen at the time of enrollment, 5 were UMs, 125 EMs, 24 IMs, 7 PMs and 5 'no genotype'.

**Table 2:** Distribution of *CYP2D6 *genotype and predicted phenotype by different ethnic groups

	Ethnicity							
		
*CYP2D6 *predicted phenotype/genotype	Caucasian	Latina/Hispanic	AA/Black	Asian	Pacific Islander	Other/mixed	Declined/missed	Total N (%)
Ultra-rapid (UM)	**8 (5%)**	**1 (7%)**	**0**	**1 (2%)**	**0**	**0**	**0**	**10 (4%)**
*1/*1 × N	5	0	0	0	0	0	0	5
*1/*2 × N	1	1	0	0	0	0	0	2
*2/*1 × N	1	0	0	1	0	0	0	2
*2/*2 × N	1	0	0	0	0	0	0	1
								
Extensive (EM)	**131 (79%)**	**9 (65%)**	**2 (100%)**	**36 (64%)**	**1 (100%)**	**3 (100%)**	**3 (100%)**	**185 (76%)**
*1/*1	19	2	1	2	1	1	0	26
*1/*2	20	0	0	5	0	0	0	25
*1/*3	2	0	0	0	0	0	0	2
*1/*4	23	1	0	1	0	0	1	26
*1/*5	3	0	0	1	0	1	0	5
*1/*6	1	0	0	0	0	0	0	1
*1/*9	4	2	0	0	0	0	0	6
*1/*10	1	0	0	15	0	0	0	16
*1/*17	1	0	1	0	0	0	0	2
*1/*29	0	0	0	0	0	0	1	1
*1/*35	2	0	0	0	0	0	0	2
*1/*41	16	1	0	1	0	0	0	18
*1 × N/*5	1	0	0	0	0	0	0	1
*1 × N/*10	0	0	0	1	0	0	0	1
*2/*2	5	0	0	0	0	0	0	5
*2/*4	11	1	0	0	0	0	1	13
*2/*5	1	0	0	0	0	0	0	1
*2/*9	1	0	0	0	0	0	0	1
*2/*10	0	1	0	10	0	0	0	11
*2/*35	3	0	0	0	0	0	0	3
*2/*41	5	0	0	0	0	0	0	5
*2/*41 × N	1	0	0	0	0	0	0	1
*2 × N/*4	1	0	0	0	0	0	0	1
*2 × N/*9	1	0	0	0	0	0	0	1
*3/*35	1	0	0	0	0	0	0	1
*4/*35	5	0	0	0	0	0	0	5
*5/*35	0	1	0	0	0	0	0	1
*10/*35	1	0	0	0	0	0	0	1
*17/*35	0	0	0	0	0	1	0	1
*35/*41	1	0	0	0	0	0	0	1
*35/*41 × N	1	0	0	0	0	0	0	1
								
Intermediate (IM)	**15 (9%)**	**2 (14%)**	**0**	**15 (27%)**	**0**	**0**	**0**	**32 (13%)**
*4/*9	1	0	0	0	0	0	0	1
*4/*10	2	0	0	0	0	0	0	2
*4/*17	1	0	0	0	0	0	0	1
*4/*41	6	1	0	0	0	0	0	7
*5/*10	1	0	0	2	0	0	0	3
*10/*10	0	0	0	13	0	0	0	13
*10/*41	2	0	0	0	0	0	0	2
*29/*41	0	1	0	0	0	0	0	1
*41/*41	2	0	0	0	0	0	0	2
								
Poor (PM)	**10 (6%)**	**2 (14%)**	**0**	**1 (2%)**	**0**	**0**	**0**	**13 (5%)**
*3/*4	1	0	0	0	0	0	0	1
*4/*4	7	0	0	1	0	0	0	8
*4/*5	0	2	0	0	0	0	0	2
*4/*6	1	0	0	0	0	0	0	1
*4/*7	1	0	0	0	0	0	0	1
								
No genotype	**2 (1%)**	**0**	**0**	**3 (5%)**	**0**	**0**	**0**	**5 (2%)**
								
Total	**166**	**14**	**2**	**56**	**1**	**3**	**3**	**245**

AA, African American; EM, extensive metabolizer; IM, intermediate metabolizer; N, number of participants in the study; PM, poor metabolizer; UM, ultra-rapid metabolizer.

We found a significant association between *CYP2D6 *phenotype results and change in therapy (Table 3). Six of the 13 PMs (46%) changed treatment to an AI, compared to 10 out of 186 in the UM/EM group (*P *< 0.001). In contrast, there was no significant difference in treatment change rates between the women classified as IMs, 1 (3%, pre-menopausal) out of 32, and UMs/EMs (*P *= 0.51). In addition, all four women with 'no genotype' call were taking tamoxifen at the time of follow-up, which was no different than the proportion of women taking tamoxifen among UMs/EMs.

**Table 3:** Association of *CYP2D6 *testing and therapeutic decision-making by *CYP2D6 *phenotypes

*CYP2D6 *phenotype	Still on tamoxifen	Changed to AIs	No therapy	Total	***P***^**a**^	Taking co-medications	Changed co-medication	***P***^**a**^
Ultra-rapid (UM)/extensive metabolizer (EM)^b^	156 (84%)	10 (5%)	20 (11%)	**186**		38 (21%)	9 (5%)	
Intermediate metabolizer (IM)	28 (88%)	1 (3%)	3 (9%)	**32**	0.51	8 (25%)	2 (3%)	0.62
Poor metabolizer (PM)	4 (31%)	6 (46%)	3 (23%)	**13**	**< 0.001**	0	0	-
Total	**188**	**17**	**26**	**231**		**46**	**11**	

^a^*P*-value based on Fisher's exact test of association versus UM/EM. ^b^Ultra-rapid metabolizer (UM) data combined with extensive metabolizer (EM) data. AI, aromatase inhibitor.

Among the subset of pre-menopausal women (*n *= 183), 5 of 11 women with the PM phenotype switched to an AI and OS, which was significantly higher (*P *= 0.001) than the rate of change among the UMs/EMs (5 of 149). There was no difference among women with the UM/EM versus IM phenotype when we analyzed the pre-menopausal women (*P *= 0.54).

A total of 26 women reported that they were not taking hormone therapy at the time of follow-up. Of these women, four (three EMs and one IM, all pre-menopausal) were considering tamoxifen for prevention, seven (six EMs and one IM) were considering tamoxifen for treatment of DCIS and six (five EMs and one IM) for treatment of invasive breast cancer. There was no difference in the probability of being on or off hormone therapy by *CYP2D6 *metabolizer status.

Of the 186 UMs/EMs, 21% (38) were taking one or more co-medications at the time of enrollment. Nine of these 38 women (24%) changed or stopped a co-medication at the time of follow-up. Of the women on the most potent inhibitors, two of nine stopped a co-medication. There was no significant difference in the rate of change of co-medication between IMs compared to UMs/EMs (*P *= 0.62). None of the PMs were taking any of the co-medications and *CYP2D6 *inhibitors described in Table 1.

We also evaluated whether any factors besides *CYP2D6 *genotype predict change in therapy (Table 4). In univariate analyses there was no association between change to AIs and method of referral or previous knowledge of *CYP2D6 *testing. Among women who said they had prior knowledge, the source of knowledge (physician versus medical literature versus internet) did not affect choice of therapy. We also found no association between change in therapy and report of interest in *CYP2D6 *testing (*P *= 0.34) or report of interest in new medical treatments and technology (*P *= 0.59). In addition, age, race/ethnicity and education were not predictive of change in therapy (results not shown). No other significant associations were found. Specifically, age, menopausal status, educational attainment, race/ethnicity and indication for treatment (invasive cancer versus carcinoma *in situ *versus prevention) did not predict change in therapy in univariate analyses. There was no association between change in hormone therapy and reported side effects from tamoxifen.

**Table 4:** Association of therapeutic decision-making by clinical and breast cancer characteristics, *CYP2D6 *phenotype, previous knowledge of *CYP2D6 *testing, referral method, and interest in *CYP2D6 *testing

	Change to aromatase inhibitors	
	
	Unadjusted	**Adjusted**^**a**^
Characteristics	OR (95% CI) *P*-value	OR (95% CI) *P*-value
Age	1.03 (0.98, 1.08) 0.23	1.02 (0.95, 1.10) 0.50
		
Breast cancer type		
Invasive breast cancer	-	-
Ductal carcinoma *in situ *(DCIS)	1.07 (0.32, 3.48) 0.90	1.33 (0.34, 5.11) 0.67
Lobular carcinoma *in situ *(LCIS)	0.82 (0.09, 6.76) 0.85	0.44 (0.03, 5.59) 0.53
		
Post-menopausal status	1.54 (0.51, 4.62) 0.43	1.78 (0.35, 9.08) 0.48
		
Report of any tamoxifen side effects (yes)	1.09 (0.34, 3.50) 0.87	0.61 (0.16, 2.31) 0.47
		
*CYP2D6 *phenotype		
UM/EM^b^	-	-
IM	0.56 (0.07, 4.59) 0.59	0.38 (0.04, 3.38) 0.38
PM	**15.08 (4.26, 53.33) 0.0001**	**22.85 (5.28, 98.74) 0.0001**
		
Previous knowledge of *CYP2D6 *testing (yes)	0.88 (0.33, 2.38) 0.81	0.91 (0.29, 2.84) 0.87
		
Referred by physician or nurse (yes)	0.60 (0.20, 1.81) 0.37	0.36 (0.09, 1.37) 0.13
		
Very interested in *CYP2D6 *testing before attending the educational session (versus somewhat and not really interested)	1.77 (0.49, 6.38) 0.38	3.01 (0.66, 13.65) 0.15

The number of participants followed up was 235. ^a^Odd ratios adjusted for age, breast cancer type, menopausal status, report of any tamoxifen side effects, *CYP2D6 *phenotype, previous knowledge of *CYP2D6 *testing, referral method, and interest in *CYP2D6 *testing. *P*-value ≤ 0.05. ^b^Ultra-rapid metabolizer (UM) data combined with extensive metabolizer (EM) data. CI, confidence interval; DCIS, ductal carcinoma *in situ*; EM, extensive metabolizer; IM, intermediate metabolizer; LCIS, lobular carcinoma *in situ*; N, number of participants; OR, odds ratio; PM, poor metabolizer; UM, ultra-rapid metabolizer.

We used multi-variate models to determine whether any factors may confound the association between *CYP2D6 *genotype and change in therapy (Table 4). *CYP2D6 *genotype remained the only statistically significant association with change in therapy even after adjustment for age, breast cancer type (invasive breast cancer, DCIS and lobular carcinoma *in situ *(LCIS)), menopausal status (pre-menopausal versus post-menopausal), report of any tamoxifen-induced side effects, previous knowledge of *CYP2D6 *testing, referral method (physician or nurse versus other sources) and interest in *CYP2D6 *testing.

## Discussion

We provided *CYP2D6 *genotype results to clinicians and patients and evaluated the impact of this information on the proportion of women who changed hormone therapy. Approximately 5% of women were PMs and 6 out of 13 (46%) changed treatment after discussion with their physicians. This was a significantly higher percentage than the rate of therapy change in those with UM, EM or IM phenotypes, suggesting that in this setting phenotype results affected treatment decisions. The association between medication change was not confounded by method of referral to the study or by prior interest in *CYP2D6 *testing.

For pre-menopausal women, change in hormone therapy included both an AI as well as ovarian suppression that leads to significant side effects associated with early menopause. Thus, despite the limited evidence and the risk of side effects from an alternative treatment, physicians and patients frequently changed therapy in response to a PM phenotype. Treatment with an AI alone in younger women who have amenorrhea due to chemotherapy may lead to inadequate hormonal suppression [40]. We did not directly make any treatment recommendations. But five of the six women who had changed to an AI also received OS. The only woman who received AI alone was aged 56 years and was known to be post-menopausal prior to breast cancer treatment. Therefore, the physicians who referred to our study appear to be aware of the risks of inadequate hormonal therapy and to have used combination therapy when appropriate.

The association between *CYP2D6 *genotype and efficacy of tamoxifen in women with early stage, hormone receptor-positive breast cancer remains unclear. *CYP2D6 *activity clearly correlates with endoxifen levels, but the association with outcome has been far more controversial. Several studies have demonstrated an association [11,14-19], but other studies, including the two largest, have failed to confirm an impact of enzyme activity and breast cancer outcome [20-23,25,26]. A recent meta-analysis found a trend towards association between *CYP2D6 *genotype and disease free survival but not overall survival [41]. However, the authors noted considerable heterogeneity among the studies in both the reported associations and in the way subsets of genotypes were grouped. More recently, two large randomized controlled trials, the Arimidex, Tamoxifen, Alone or in Combination (ATAC) trial [25] and the Breast International Group (BIG) 1-98 trial [26], evaluated the impact of *CYP2D6 *polymorphisms in patients treated with tamoxifen. Neither study demonstrated an association between the risk of breast cancer recurrence and *CYP2D6 *phenotype.

Our study had completed all the enrollment and the follow-up by September 2010, prior to the presentation of the genotyping data from the ATAC and BIG 1-98 trials in December 2010. Thus, at the time that patients and clinicians were deciding how to interpret the genotypes, these results could not be taken into consideration, but could have a significant impact on decisions regarding testing and treatment change. However, as part of our presentation to patients prior to testing, we showed the results of both prior positive and negative studies testing for associations between *CYP2D6 *and breast cancer. Since physicians and patients were aware of the controversial results, our study demonstrates that many clinicians and patients are generally willing to make treatment decisions even in the curative setting based on non-definitive and retrospective pharmacogenetic information when accompanied by a reasonable hypothesis.

The prevalence of *CYP2D6 *polymorphisms varies across ethnic groups. The frequency of *CYP2D6 *PMs in our study is consistent with previous reports [15,19]. Like other investigators [14,24,42], we found that the most frequent variant present in Asians was CYP2D6*10, an allele with reduced activity. Results examining the association between the *10 allele and clinical outcomes have also been mixed [14,16,19,20,24,42]. The rate of medication change among patients with the IM *CYP2D6 *phenotype, including patients homozygous for this allele, was similar to that in patients with the UM/EM phenotype, suggesting that physicians do not consider these patients at significantly increased risk of recurrence.

Endoxifen concentration varies not only according to the number of functional *CYP2D6 *alleles [43] but also in the presence of potent *CYP2D6 *enzyme inhibitors. Agents such as the SSRIs paroxetine or fluoxetine, and the anti-arrhythmic quinidine are among the most potent inhibitors [9,44]. When these medications are co-administered with tamoxifen to women with an EM phenotype, endoxifen concentrations are similar to those observed in PM and have the potential, therefore, to reduce tamoxifen efficacy [9,43,44]. Other commonly used medications such as buproprion, duloxetine, clomipramine, thioridazine, pherphenazine, and pimozide exhibit inhibition close to that of paroxetine, fluoxetine and quinidine [44-46]. While we found that some women did change their co-medications, this was unrelated to *CYP2D6 *genotype. Our study did not collect enough information from physicians to distinguish between those two possibilities.

Our study is unique in that, to our knowledge, no prior pharmacogenetic studies on change in therapy for *CYP2D6 *have been previously published. Several studies have examined the issue of incorporating pharmacogenetic data in dosing warfarin [47,48]; however, genetic testing for warfarin dosing does not involve a change to a different medication. Several studies have also shown that genetic testing for *BRCA1*/*2 *leads to selection of risk-reducing surgeries [49-51], the use of post-menopausal hormone therapy [52], and pre-implantation genetic diagnosis [53].

Our study also has several important limitations. First, the evidence for the association between *CYP2D6 *polymorphisms and outcomes remains mixed in the literature and the availability of the most recent results may have changed the decisions that patients and providers in our study made. Second, our sample may have been biased by referral patterns and by patient participation. Physicians and patients who are interested in testing and in changing therapy based on test results may have been more likely to participate in our study. However, we found no association between prior knowledge or interest in *CYP2D6 *genotype testing and choice of therapy at follow-up. In addition, there were no other significant predictors within our data. Third, our sample may not be universally generalizable. Our patients tended to be mostly Caucasians and Asians, highly educated on average, with a relatively high income level, and most were already being followed at a University medical center for breast cancer. Furthermore, our study used patient self-report of medication use rather than chart review or physician report. However, both patient report and physician report may have limitations. More studies should be conducted to determine how genotyping results would be used in community settings.

## Conclusions

Our study demonstrates that *CYP2D6 *pharmacogenetic testing led to change in therapy among patients with genotypes that predicted no *CYP2D6 *activity. Thus, clinicians and patients do use pharmacogenetic results to change therapy, even in the absence of definitive knowledge about the utility of the pharmacogenetic result. Ultimately, prospective randomized trials will be required to demonstrate the impact of treatment change based on pharmacogenetic testing.

## Abbreviations

AI: aromatase inhibitor; ATAC: Arimidex, Tamoxifen, Alone or in Combination; BIG: Breast International Group; *CYP2C19*: cytochrome P450 2C19; *CYP2D6*: cytochrome P450 2D6; DCIS: ductal carcinoma *in situ*; EM: extensive metabolizer; IM: intermediate metabolizer; OS: ovarian suppression; PM: poor metabolizer; SSRI: selective serotonin reuptake inhibitor; UCSF: University of California San Francisco; UM: ultra-rapid metabolizer.

## Competing interests

HJL and MN are full-time employees of Roche Molecular Systems, Inc., which manufactures the AmpliChip CYP450 Test. These authors were not involved in any of the presentations of information to patients regarding the assay either prior to or after testing. They were involved in assisting the UCSF investigators with technical questions regarding the assay, and were involved in critical revisions of the manuscript. The rest of the authors declare that they have no competing interests.

## Authors' contributions

WL and EZ participated in the conception, design and implementation of the study, acquisition of data, performed the statistical analysis and interpretation of data, and participated in drafting and preparation of the manuscript. HR, MSB, and MM participated in the conception of the study, acquisition of data and drafting the manuscript. TM provided administrative and institutional support, and participated in drafting the manuscript. ST and AHBW carried out the DNA extraction, *CYP2D6 *genotype assay and interpretation, and participated in drafting the manuscript. HJL and MN provided technical support and interpretation for the AmpliChip CYP450 Test and Data Analysis Software. All authors read and approved the final version of the manuscript.

## References

[B1] Pirmohamed M Acceptance of biomarker-based tests for application in clinical practice: criteria and obstacles. Clin Pharmacol Ther 2010 88 862 866 10.1038/clpt.2010.245 20981006

[B2] Kitzmiller JP Groen DK Phelps MA Sadee W Pharmacogenomic testing: relevance in medical practice: why drugs work in some patients but not in others. Cleve Clin J Med 2011 78 243 257 10.3949/ccjm.78a.10145 21460130 PMC3351041

[B3] Wong WB Carlson JJ Thariani R Veenstra DL Cost effectiveness of pharmacogenomics: a critical and systematic review. Pharmacoeconomics 2010 28 1001 1013 10.2165/11537410-000000000-00000 20936884

[B4] Jordan VC Tamoxifen: a most unlikely pioneering medicine. Nat Rev Drug Discov 2003 2 205 213 10.1038/nrd1031 12612646

[B5] Group EBCTC Effects of chemotherapy and hormonal therapy for early breast cancer on recurrence and 15-year survival: an overview of the randomised trials. Lancet 2005 365 1687 1717 15894097 10.1016/S0140-6736(05)66544-0

[B6] Group EBCTC Tamoxifen for early breast cancer: an overview of randomized trials. Lancet 1998 351 1451 1467 9605801

[B7] Fisher B Costantino JP Wickerham DL Redmond CK Kavanah M Cronin WM Vogel V Robidoux A Dimitrov N Atkins J Daly M Wieand S Tan-Chiu E Ford L Wolmark N Tamoxifen for prevention of breast cancer: report of the National Surgical Adjuvant Breast and Bowel Project P-1 Study. J Natl Cancer Inst 1998 90 1371 1388 10.1093/jnci/90.18.1371 9747868

[B8] Lien EA Solheim E Lea OA Lundgren S Kvinnsland S Ueland PM Distribution of 4-hydroxy-N-desmethyltamoxifen and other tamoxifen metabolites in human biological fluids during tamoxifen treatment. Cancer Res 1989 49 2175 2183 2702659

[B9] Stearns V Johnson MD Rae JM Morocho A Novielli A Bhargava P Hayes DF Desta Z Flockhart DA Active tamoxifen metabolite plasma concentrations after coadministration of tamoxifen and the selective serotonin reuptake inhibitor paroxetine. J Natl Cancer Inst 2003 95 1758 1764 14652237 10.1093/jnci/djg108

[B10] Wu X Hawse JR Subramaniam M Goetz MP Ingle JN Spelsberg TC The tamoxifen metabolite, endoxifen, is a potent antiestrogen that targets estrogen receptor alpha for degradation in breast cancer cells. Cancer Res 2009 69 1722 1727 10.1158/0008-5472.CAN-08-3933 19244106

[B11] Jin Y Desta Z Stearns V Ward B Ho H Lee KH Skaar T Storniolo AM Li L Araba A Blanchard R Nguyen A Ullmer L Hayden J Lemler S Weinshilboum RM Rae JM Hayes DF Flockhart DA CYP2D6 genotype, antidepressant use, and tamoxifen metabolism during adjuvant breast cancer treatment. J Natl Cancer Inst 2005 97 30 39 10.1093/jnci/dji005 15632378

[B12] Jordan VC Collins MM Rowsby L Prestwich G A monohydroxylated metabolite of tamoxifen with potent antioestrogenic activity. J Endocrinol 1977 75 305 316 10.1677/joe.0.0750305 591813

[B13] Desta Z Ward BA Soukhova NV Flockhart DA Comprehensive evaluation of tamoxifen sequential biotransformation by the human cytochrome P450 system in vitro: prominent roles for CYP3A and CYP2D6. J Pharmacol Exp Ther 2004 310 1062 1075 10.1124/jpet.104.065607 15159443

[B14] Xu Y Sun Y Yao L Shi L Wu Y Ouyang T Li J Wang T Fan Z Fan T Lin B He L Li P Xie Y Association between CYP2D6 *10 genotype and survival of breast cancer patients receiving tamoxifen treatment. Ann Oncol 2008 19 1423 1429 10.1093/annonc/mdn155 18407954

[B15] Bijl MJ van Schaik RH Lammers LA Hofman A Vulto AG van Gelder T Stricker BH Visser LE The CYP2D6*4 polymorphism affects breast cancer survival in tamoxifen users. Breast Cancer Res Treat 2009 118 125 130 10.1007/s10549-008-0272-2 19189212

[B16] Kiyotani K Mushiroda T Sasa M Bando Y Sumitomo I Hosono N Kubo M Nakamura Y Zembutsu H Impact of CYP2D6*10 on recurrence-free survival in breast cancer patients receiving adjuvant tamoxifen therapy. Cancer Sci 2008 99 995 999 10.1111/j.1349-7006.2008.00780.x 18294285 PMC11158376

[B17] Schroth W Antoniadou L Fritz P Schwab M Muerdter T Zanger UM Simon W Eichelbaum M Brauch H Breast cancer treatment outcome with adjuvant tamoxifen relative to patient CYP2D6 and CYP2C19 genotypes. J Clin Oncol 2007 25 5187 5193 10.1200/JCO.2007.12.2705 18024866

[B18] Goetz MP Knox SK Suman VJ Rae JM Safgren SL Ames MM Visscher DW Reynolds C Couch FJ Lingle WL Weinshilboum RM Fritcher EG Nibbe AM Desta Z Nguyen A Flockhart DA Perez EA Ingle JN The impact of cytochrome P450 2D6 metabolism in women receiving adjuvant tamoxifen. Breast Cancer Res Treat 2007 101 113 121 10.1007/s10549-006-9428-0 17115111

[B19] Lim HS Ju Lee H Seok Lee K Sook Lee E Jang IJ Ro J Clinical implications of CYP2D6 genotypes predictive of tamoxifen pharmacokinetics in metastatic breast cancer. J Clin Oncol 2007 25 3837 3845 10.1200/JCO.2007.11.4850 17761971

[B20] Okishiro M Taguchi T Jin Kim S Shimazu K Tamaki Y Noguchi S Genetic polymorphisms of CYP2D6*10 and CYP2C19*2, *3 are not associated with prognosis, endometrial thickness, or bone mineral density in Japanese breast cancer patients treated with adjuvant tamoxifen. Cancer 2009 115 952 961 10.1002/cncr.24111 19156902

[B21] Wegman P Elingarami S Carstensen J Stal O Nordenskjold B Wingren S Genetic variants of CYP3A5, CYP2D6, SULT1A1, UGT2B15 and tamoxifen response in postmenopausal patients with breast cancer. Breast Cancer Res 2007 9 R7 10.1186/bcr1640 17244352 PMC1851378

[B22] Wegman P Vainikka L Stal O Nordenskjold B Skoog L Rutqvist LE Wingren S Genotype of metabolic enzymes and the benefit of tamoxifen in postmenopausal breast cancer patients. Breast Cancer Res 2005 7 R284 290 10.1186/bcr993 15987423 PMC1143572

[B23] Nowell SA Ahn J Rae JM Scheys JO Trovato A Sweeney C MacLeod SL Kadlubar FF Ambrosone CB Association of genetic variation in tamoxifen-metabolizing enzymes with overall survival and recurrence of disease in breast cancer patients. Breast Cancer Res Treat 2005 91 249 258 10.1007/s10549-004-7751-x 15952058

[B24] Toyama T Yamashita H Sugiura H Kondo N Iwase H Fujii Y No association between CYP2D6*10 genotype and survival of node-negative Japanese breast cancer patients receiving adjuvant tamoxifen treatment. Jpn J Clin Oncol 2009 39 651 656 10.1093/jjco/hyp076 19596663

[B25] Rae JM Drury S Hayes DF Stearns V Thibert JN Haynes BP Salter J Pineda S Cuzick J Dowsett M Lack of correlation between gene variants in tamoxifen metabolizing enzymes with primary endpoints in the ATAC trial [abstract]. Cancer Res 2010 70 Suppl 2 nr S1 7

[B26] Leyland-Jones B Regan MM Bouzyk M Kammler R Tang W Pagani O Maibach R Dell'Orto P Thurlimann B Price KN Viale G Group. B-CGaIBCS Outcome according to CYP2D6 genotype among postmenopausal women with endocrine-responsive early invasive breast cancer randomized in the BIG 1-98 trial [abstract]. Cancer Res 2010 70 Suppl 2 nr S1 8

[B27] Cuzick J Sestak I Baum M Buzdar A Howell A Dowsett M Forbes JF Effect of anastrozole and tamoxifen as adjuvant treatment for early-stage breast cancer: 10-year analysis of the ATAC trial. Lancet Oncol 2010 11 1135 1141 10.1016/S1470-2045(10)70257-6 21087898

[B28] Colleoni M Giobbie-Hurder A Regan MM Thurlimann B Mouridsen H Mauriac L Forbes JF Paridaens R Lang I Smith I Chirgwin J Pienkowski T Wardley A Price KN Gelber RD Coates AS Goldhirsch A Analyses adjusting for selective crossover show improved overall survival with adjuvant letrozole compared with tamoxifen in the BIG 1-98 study. J Clin Oncol 2011 29 1117 1124 10.1200/JCO.2010.31.6455 21321298 PMC3083867

[B29] Li CI Daling JR Malone KE Incidence of invasive breast cancer by hormone receptor status from 1992 to 1998. J Clin Oncol 2003 21 28 34 10.1200/JCO.2003.03.088 12506166

[B30] Jassem J Intergroup Exemestane Study mature analysis: overall survival data. Anticancer Drugs 2008 19 Suppl 1 S3 7 18340242 10.1097/01.cad.0000277608.23376.bc

[B31] Howell A Cuzick J Baum M Buzdar A Dowsett M Forbes JF Hoctin-Boes G Houghton J Locker GY Tobias JS Results of the ATAC (Arimidex, Tamoxifen, Alone or in Combination) trial after completion of 5 years' adjuvant treatment for breast cancer. Lancet 2005 365 60 62 15639680 10.1016/S0140-6736(04)17666-6

[B32] Thurlimann B Keshaviah A Coates AS Mouridsen H Mauriac L Forbes JF Paridaens R Castiglione-Gertsch M Gelber RD Rabaglio M Smith I Wardley A Price KN Goldhirsch A A comparison of letrozole and tamoxifen in postmenopausal women with early breast cancer. N Engl J Med 2005 353 2747 2757 16382061 10.1056/NEJMoa052258

[B33] Goss PE Ingle JN Martino S Robert NJ Muss HB Piccart MJ Castiglione M Tu D Shepherd LE Pritchard KI Livingston RB Davidson NE Norton L Perez EA Abrams JS Cameron DA Palmer MJ Pater JL Randomized trial of letrozole following tamoxifen as extended adjuvant therapy in receptor-positive breast cancer: updated findings from NCIC CTG MA.17. J Natl Cancer Inst 2005 97 1262 1271 10.1093/jnci/dji250 16145047

[B34] Colleoni M Gelber S Goldhirsch A Aebi S Castiglione-Gertsch M Price KN Coates AS Gelber RD Tamoxifen after adjuvant chemotherapy for premenopausal women with lymph node-positive breast cancer: International Breast Cancer Study Group Trial 13-93. J Clin Oncol 2006 24 1332 1341 16505417 10.1200/JCO.2005.03.0783

[B35] Cuzick J Ambroisine L Davidson N Jakesz R Kaufmann M Regan M Sainsbury R Use of luteinising-hormone-releasing hormone agonists as adjuvant treatment in premenopausal patients with hormone-receptor-positive breast cancer: a meta-analysis of individual patient data from randomised adjuvant trials. Lancet 2007 369 1711 1723 17512856 10.1016/S0140-6736(07)60778-8

[B36] Gnant M Mlineritsch B Schippinger W Luschin-Ebengreuth G Postlberger S Menzel C Jakesz R Seifert M Hubalek M Bjelic-Radisic V Samonigg H Tausch C Eidtmann H Steger G Kwasny W Dubsky P Fridrik M Fitzal F Stierer M Rucklinger E Greil R Marth C Endocrine therapy plus zoledronic acid in premenopausal breast cancer. N Engl J Med 2009 360 679 691 10.1056/NEJMoa0806285 19213681

[B37] Puhalla S Brufsky A Davidson N Adjuvant endocrine therapy for premenopausal women with breast cancer. Breast 2009 18 Suppl 3 S122 130 19914530 10.1016/S0960-9776(09)70286-3 PMC3901991

[B38] Rebsamen MC Desmeules J Daali Y Chiappe A Diemand A Rey C Chabert J Dayer P Hochstrasser D Rossier MF The AmpliChip CYP450 test: cytochrome P450 2D6 genotype assessment and phenotype prediction. Pharmacogenomics J 2009 9 34 41 10.1038/tpj.2008.7 18591960

[B39] Flockhart DA Drug Interactions: Cytochrome P450 Drug Interaction Table. Indiana University School of Medicine. http://medicine.iupui.edu/clinpharm/ddis/table.aspx

[B40] Smith IE Dowsett M Yap YS Walsh G Lonning PE Santen RJ Hayes D Adjuvant aromatase inhibitors for early breast cancer after chemotherapy-induced amenorrhoea: caution and suggested guidelines. J Clin Oncol 2006 24 2444 2447 10.1200/JCO.2005.05.3694 16735701

[B41] Seruga B Amir E Cytochrome P450 2D6 and outcomes of adjuvant tamoxifen therapy: results of a meta-analysis. Breast Cancer Res Treat 2010 122 609 617 10.1007/s10549-010-0902-3 20454926

[B42] Kiyotani K Mushiroda T Imamura CK Hosono N Tsunoda T Kubo M Tanigawara Y Flockhart DA Desta Z Skaar TC Aki F Hirata K Takatsuka Y Okazaki M Ohsumi S Yamakawa T Sasa M Nakamura Y Zembutsu H Significant effect of polymorphisms in CYP2D6 and ABCC2 on clinical outcomes of adjuvant tamoxifen therapy for breast cancer patients. J Clin Oncol 2010 28 1287 1293 10.1200/JCO.2009.25.7246 20124171 PMC4872305

[B43] Borges S Desta Z Li L Skaar TC Ward BA Nguyen A Jin Y Storniolo AM Nikoloff DM Wu L Hillman G Hayes DF Stearns V Flockhart DA Quantitative effect of CYP2D6 genotype and inhibitors on tamoxifen metabolism: implication for optimization of breast cancer treatment. Clin Pharmacol Ther 2006 80 61 74 10.1016/j.clpt.2006.03.013 16815318

[B44] Sideras K Ingle JN Ames MM Loprinzi CL Mrazek DP Black JL Weinshilboum RM Hawse JR Spelsberg TC Goetz MP Coprescription of tamoxifen and medications that inhibit CYP2D6. J Clin Oncol 2010 28 2768 2776 10.1200/JCO.2009.23.8931 20439629 PMC2881853

[B45] Skinner MH Kuan HY Pan A Sathirakul K Knadler MP Gonzales CR Yeo KP Reddy S Lim M Ayan-Oshodi M Wise SD Duloxetine is both an inhibitor and a substrate of cytochrome P4502D6 in healthy volunteers. Clin Pharmacol Ther 2003 73 170 177 10.1067/mcp.2003.28 12621382

[B46] Kotlyar M Brauer LH Tracy TS Hatsukami DK Harris J Bronars CA Adson DE Inhibition of CYP2D6 activity by bupropion. J Clin Psychopharmacol 2005 25 226 229 10.1097/01.jcp.0000162805.46453.e3 15876900

[B47] Hill CE Duncan A Overview of pharmacogenetics in anticoagulation therapy. Clin Lab Med 2008 28 513 524 10.1016/j.cll.2008.09.002 19059059

[B48] Mahajan P Meyer KS Wall GC Price HJ Clinical applications of pharmacogenomics guided warfarin dosing. Int J Clin Pharm 2011 33 10 19 10.1007/s11096-011-9486-1 21365388

[B49] Evans DG Lalloo F Ashcroft L Shenton A Clancy T Baildam AD Brain A Hopwood P Howell A Uptake of risk-reducing surgery in unaffected women at high risk of breast and ovarian cancer is risk, age, and time dependent. Cancer Epidemiol Biomarkers Prev 2009 18 2318 2324 10.1158/1055-9965.EPI-09-0171 19661091

[B50] Domchek SM Friebel TM Singer CF Evans DG Lynch HT Isaacs C Garber JE Neuhausen SL Matloff E Eeles R Pichert G Van t'veer L Tung N Weitzel JN Couch FJ Rubinstein WS Ganz PA Daly MB Olopade OI Tomlinson G Schildkraut J Blum JL Rebbeck TR Association of risk-reducing surgery in BRCA1 or BRCA2 mutation carriers with cancer risk and mortality. JAMA 2010 304 967 975 10.1001/jama.2010.1237 20810374 PMC2948529

[B51] Beattie MS Crawford B Lin F Vittinghoff E Ziegler J Uptake, time course, and predictors of risk-reducing surgeries in BRCA carriers. Genet Test Mol Biomarkers 2009 13 51 56 10.1089/gtmb.2008.0067 19309274 PMC2981359

[B52] Gabriel CA Tigges-Cardwell J Stopfer J Erlichman J Nathanson K Domchek SM Use of total abdominal hysterectomy and hormone replacement therapy in BRCA1 and BRCA2 mutation carriers undergoing risk-reducing salpingo-oophorectomy. Fam Cancer 2009 8 23 28 10.1007/s10689-008-9208-6 18758995

[B53] Quinn GP Vadaparampil ST McGowan Lowrey K Eidson S Knapp C Bukulmez O State laws and regulations addressing third-party reimbursement for infertility treatment: implications for cancer survivors. Fertil Steril 2011 95 72 78 10.1016/j.fertnstert.2010.05.017 20576264

